# Willingness to participate in pragmatic dialysis trials: the importance of physician decisional autonomy and consent approach

**DOI:** 10.1186/s13063-017-2217-8

**Published:** 2017-10-11

**Authors:** Katherine R. Courtright, Scott D. Halpern, Steven Joffe, Susan S. Ellenberg, Jason Karlawish, Vanessa Madden, Nicole B. Gabler, Stephanie Szymanski, Kuldeep N. Yadav, Laura M. Dember

**Affiliations:** 1 Division of Pulmonary, Allergy, and Critical Care Medicine, Department of Medicine, University of Pennsylvania, Philadelphia, PA USA; 2 Leonard Davis Institute of Health Economics, University of Pennsylvania, Philadelphia, PA USA; 3 Department of Biostatistics, Epidemiology and Informatics, University of Pennsylvania, Philadelphia, PA USA; 4 Department of Medical Ethics and Health Policy, University of Pennsylvania, Philadelphia, PA USA; 5 Division of Oncology, Children’s Hospital of Philadelphia, Philadelphia, PA USA; 6 Division of Geriatrics, Department of Medicine, University of Pennsylvania, Philadelphia, PA USA; 7 Renal-Electrolyte and Hypertension Division, Department of Medicine, University of Pennsylvania, Philadelphia, PA USA

**Keywords:** Pragmatic clinical trial, Ethics, Informed consent, Autonomy, Standard of care, Comparative effectiveness research

## Abstract

**Background:**

Pragmatic clinical trials embedded in routine delivery of clinical care can lead to improvements in quality of care, but often have design features that raise ethical concerns.

**Methods:**

We performed a discrete choice experiment and used conjoint analysis to assess how specific attributes of pragmatic dialysis trials influenced patients’ and physicians’ willingness to have their dialysis facility participate in a hypothetical trial of hypertension management. Electronic survey data were collected from 200 patients enrolled from 11 outpatient hemodialysis units and from 203 nephrologists. The three attributes studied were physicians’ treatment autonomy, participants’ research burden, and the approach to consent. The influence of each attribute was quantified using mixed-effects logistic regression.

**Results:**

Similar proportions of patients were willing to have their facilities participate in a trial with high vs. low physician autonomy (77% vs. 79%; *p* = 0.13) and research burden (76% vs. 80%; *p* = 0.06). Opt-in, opt-out, and notification-only consent approaches were acceptable to most patients (84%, 82%, and 81%, respectively), but compared to each of these consent approaches, fewer patients (66%) were willing to have their facility participate in a trial that used no notification (*p* < 0.001 for each 2-way comparison). Among the physicians, similar proportions were willing to participate in trials with high and low physician autonomy (61% and 61%, respectively, *p* = 0.96) or with low and high burden (60 and 61%, respectively, *p* = 0.79). However, as for the patients, the consent approach influenced trial acceptability with 77%, 69%, and 62% willing to participate using opt-in, opt-out, and notification-only, respectively, compared to no notification (36%) (*p* < 0.001 for each 2-way comparison).

**Conclusions:**

Curtailing physician’s treatment autonomy and increasing the burden associated with participation did not influence patients’ or physicians’ willingness to participate in the hypothetical research, suggesting that pragmatic dialysis trials are generally acceptable to patients and physicians. Both patients and physicians preferred consent approaches that include at least some level of patient notification, but the majority of patients were still willing to participate in trials that did not notify patients of the research.

**Electronic supplementary material:**

The online version of this article (doi:10.1186/s13063-017-2217-8) contains supplementary material, which is available to authorized users.

## Background

In contrast to traditional randomized clinical trials (RCTs) that are designed to evaluate the efficacy and safety of a drug or intervention under ideal conditions, pragmatic trials aim to compare the effectiveness, benefits, and harms of interventions under real-world conditions [1]. Pragmatic trials embedded within usual clinical care delivery, or “learning health systems,” are designed to maximize the external validity of the knowledge they generate [2–4]. By facilitating rapid enrollment of large numbers of patients, pragmatic trials can markedly reduce recruitment challenges and high costs that characterize many traditional RCTs [5–15]. However, the benefits of trials embedded in clinical care must be considered in the context of the loss of patient and physician autonomy that they may impose especially if entire hospitals or clinics are randomized as a cluster to a specific treatment approach. Moreover, such trials may be conducted with waivers of informed consent. Several ethicists have noted that standard informed consent procedures may not be applicable in pragmatic trials comparing interventions that fall within the “standard of care” [16–19] because such trials do not increase risk to patients beyond the risk of usual care. However, such trials alter the process by which an individual’s care is selected, thereby limiting patients’ and physicians’ decisional autonomy [20].

As promising as pragmatic trials may be for improving the quality and costs of care [2], little empirical work has addressed these ethical concerns [17, 21–23]. In a recent qualitative study, we examined how patients and physicians value physician decisional autonomy and traditional informed consent in the context of pragmatic trials [24]. In that study, patients receiving maintenance dialysis generally accepted restrictions on physician decisional autonomy and, when necessary to achieve the goals of the study, endorsed simple notification rather than formal consent. Physicians generally expressed similar views, though some expressed greater reservations about forgoing opt-in consent [24]. Informed by these qualitative results, we designed the current study to quantify how strongly patients and physicians value physicians’ treatment autonomy relative to other desirable study features such as minimization of participant burden and opportunity for informed consent. We conducted a survey-based discrete choice study among patients with end-stage renal disease (ESRD) receiving maintenance hemodialysis and with physicians to determine the relative influence of these study attributes on willingness to participate in a pragmatic dialysis trial.

## Methods

### Setting and participants

Patients receiving maintenance hemodialysis for ESRD were recruited from 11 outpatient dialysis facilities in the greater Philadelphia region between June 2015 and March 2016. Exclusion criteria included age <18 years, initiation of dialysis within the previous 90 days, inability to speak English, and inadequate visual or cognitive function as determined by each facility’s charge nurse. Potential participants were approached during a dialysis session by a trained research coordinator. After providing informed consent, participants completed the survey using a tablet computer. Patient participants received compensation of US$10 after survey completion.

Physician members of the American Society of Nephrology (ASN) were invited to participate via email between July 2015 and February 2016. Eligible physicians were practicing nephrologists treating adult patients in outpatient dialysis units in the USA. Potentially eligible physicians were randomly selected from the ASN membership list and sent email invitations in two separate waves starting in July and December 2015, respectively, followed by two reminder emails over a 4-week period. Physician participants received a US$50 gift card for completing the study. The study was approved by DaVita Clinical Research (#18-2015) and the Institutional Review Board of the University of Pennsylvania (#819593).

### Survey instrument and study design

The survey consisted of a presentation of scenarios, each describing a hypothetical cluster-randomized clinical trial comparing two approaches to blood pressure management for patients receiving maintenance hemodialysis. The basic trial design was held constant across the scenarios but trial attributes were varied. The survey content was informed by our previous qualitative study in which semi-structured interviews were used to understand views of patients and physicians about protocolized treatment approaches for clinical trials [24]. We chose two of the three attributes evaluated in this study based on themes from these interviews: (1) the effect of protocolized care on physician treatment autonomy (treatment autonomy); and (2) information exchange between patients and physicians as it relates to the mechanism of consent (consent approach). Although the third attribute, participant burden, did not emerge as a major theme in the qualitative study, most patients and physicians interviewed did express concern about the burden of participation, making it appropriate for further evaluation. For the treatment autonomy attribute, we varied the physician’s role in selecting a blood pressure target for the patient and choosing which anti-hypertensive medications to use. For the participant burden attribute we varied the testing required for the trial from routine non-invasive blood pressure measurement to monthly blood draws and an additional research visit separate from dialysis treatment. For the consent approach attribute we varied the trial’s enrollment mechanism (opt-in consent, opt-out consent, notification-only, no-notification) (Additional file 1).

A full factorial design using these three attributes produced 16 scenarios (2 autonomy levels × 2 burden levels × 4 consent levels): however, we restricted the number of scenarios to 8 per participant by randomly assigning each to receive one of six groups of 8 scenarios to minimize participant fatigue [25]. In each group, two of the four consent approaches were tested across the two levels of autonomy and two levels of burden. The 6 groups entailed all possible pairwise comparisons of the four consent attributes. We pilot-tested the computerized presentations with 5 patients and 14 physicians to assess acceptability of the format and language, and modified them accordingly. The final survey was written at an 8th-grade literacy level and supplemented with cartoon illustrations to promote readability.

The scenarios for physicians contained more medical terminology than those directed to patients (Additional file 1: Appendix A) but had comparable content. Physicians were informed that oversight of this large, adequately powered hypothetical trial would be provided by Institutional Review Boards and an independent Data and Safety Monitoring Board, and that patients would be provided with the following statement: *Patients will not be included in the study if their doctor thinks they should not participate*.

The sequence for the eight scenarios in each group was determined by electronic randomization to mitigate potential ordering effects. After reviewing each scenario, participants used a 4-point scale to indicate willingness to have their dialysis facility participate in the clinical trial (definitely participate, probably participate, probably not participate, definitely not participate). An a priori decision was made to dichotomize willingness to participate into either a “Yes” (definitely or probably participate) or “No” (probably not or definitely not participate) for the primary analysis.

Because of limited prior research exposure among this patient population [24], patients were asked to view a 3-minute animated video entitled “Approaches to Research in Medical Practice” from the Research on Medical Practices (ROMP) Ethics Study (rompethics.iths.org) prior to presentation of the clinical trial scenarios. Patients then completed a 5-item knowledge test about randomization and standard-of-care research, with the correct answers revealed afterwards (Additional file 1: Appendix B).

After completion of the scenarios, patients completed the Research Attitude Questionnaire [26], a 7-item measure that assesses patients’ support for and value of biomedical research (theoretical scores range from 7 to 35, with higher scores indicating more favorable views). Patients also completed the 9-item Revised Health Care System Distrust Scale [27] to capture their trust in the values and competence of the healthcare system (theoretical scores range 9 to 45, with higher scores indicating more distrust). Finally, we collected demographic information about patients (age, gender, race, ethnicity, employment status, income, comorbidities, hospitalizations in the past year, and years receiving dialysis therapy) and physicians (age, gender, race, ethnicity, years in practice, practice setting, and past experience with clinical trials).

### Statistical analysis

The primary outcome, willingness to have the dialysis facility participate in the trial, was analyzed using hierarchical random-effects logistic regression with an exchangeable working correlation structure and robust error variance estimators to account for the clustering of the responses by each participant [28, 29]. Prior to model building, we examined all continuous variables using locally weighted scatterplot smoothing to determine the appropriateness of entering such variables as linear terms. No variable required transformation. The associations of participant characteristics with willingness to participate were assessed using *t* tests and Chi-square tests for continuous and categorical variables, respectively. We decided a priori to include all three trial attributes (exposures) in the baseline models for both patients and physicians. Other variables were added to the model if they had a *p* value <0.20 for the relationship between the variable and the primary outcome in a model that also included the three exposure variables. A priori we decided to also run an adjusted model without including either the healthcare system distrust or research attitude score. These data are not presented here as the results did not differ from the primary model. For physicians, none of the demographic variables met the inclusion criterion, thus the final model includes only the three trial attributes. We additionally explored potential interactions among all three trial attributes by adding two-way interaction terms one at a time to the final models for both patient and physician participants.

We based our sample size estimate on the number of trial attributes tested, the number of levels of each of these attributes, and the desire to detect an odds ratio (OR) of 0.67 for a main effect of an attribute or an OR of 0.5 for an interaction (between any two trial attributes) on the willingness to participate. We targeted 200 patients and 200 physicians for enrollment, yielding > 80% power to declare significance at *p* = 0.05. This calculation assumed that the “design effect,” [30] reflecting the number of responses per participant and the correlation of responses within participants, would be no greater than 6. The design effect is calculated as:

$$ 1+\uprho \left(\mathrm{k}-1\right), $$

where *ρ* is the intraclass correlation and *k* is the number of scenarios per participant [31].

For all analyses, two-sided *p* values <0.05 were considered statistically significant. Statistical analyses were conducted in Stata (v14.1, StataCorp, College Station, TX, USA) and R Studio (v1.0.136, RStudio Inc., Boston, MA, USA) using the R language (v1.0.136, R Foundation for Statistical Computing, Vienna, Austria) and the R package ggplot2 (v2.2.1, Springer-Verlag, New York, NY, USA).

## Results

### Patients

A total of 807 patients receiving hemodialysis therapy in 11 dialysis facilities were screened. Of these, 157 (19.4%) did not meet eligibility criteria, and 185 (22.9%) were not approached because they were absent or sleeping during attempts to enroll during two or more dialysis sessions. Among the 465 eligible patients approached, 210 (45.2%) consented to participate and 200 (43.0%) completed the entire survey.

Characteristics of the patient participants are provided in Table 1. Most were 50 years or older, male, and African American, and slightly more than half had been receiving dialysis for 3 or more years. Compared to the overall dialysis population in the geographic region, our population had a similar median age and gender profile, but a different racial distribution reflecting the predominantly urban locations of the enrolling facilities [32]. Overall, 85.0% of patient participants correctly responded to at least four of the five pre-test questions of research knowledge (Additional file 1: Appendix B). The median score of 23 (interquartile range 18.5–27) on the overall healthcare system distrust scale was consistent with scores among other outpatient populations in the same region [33].

**Table 1:** Patient characteristics

Characteristic	Patients (*N* = 200)^a^
Sex	
Male	107 (54%)
Female	93 (47%)
Age (years)	
18–29	6 (3%)
30–-39	18 (9%)
40–49	25 (13%)
50–59	44 (22%)
60–69	64 (32%)
Over 70	43 (22%)
Race	
White	67 (34%)
Black	123 (62%)
Asian	4 (2%)
Native American	5 (3%)
Hawaiian	1 (<1%)
Ethnicity	
Hispanic	9 (5%)
Not Hispanic	191 (96%)
Employment status	
Full time	10 (5%)
Part time	17 (9%)
Retired	83 (42%)
Not employed	90 (45%)
Education	
Less than high school	17 (9%)
High school	79 (40%)
Some college	56 (28%)
College degree	33 (17%)
Graduate school	15 (8%)
Annual household income	
Less than US$20,000	59 (30%)
US$20,000–US$39,999	53 (27%)
US$40,000–US$59,999	36 (18%)
US$60,000–US$79,999	23 (12%)
Over US$80,000	25 (13%)
Years on dialysis	
Less than 1 year	35 (18%)
1–3 years	59 (30%)
3–5 years	48 (24%)
More than 5 years	58 (29%)
Hospitalizations in past year	
None	62 (31%)
1	35 (18%)
2	40 (20%)
3–5	51 (26%)
More than 5	12 (6%)
Medical conditions	
Hypertension	171 (91%)
Heart disease	84 (45%)
Diabetes	84 (45%)
Chronic pulmonary diseases	23 (12%)
Malignancy	18 (10%)
Research Attitude Questionnaire^b^	28 (25–30)
Revised Healthcare System Distrust Scale^c^	23 (18.5–27)

^a^Data are presented as number (percentage) and median (IQR) for categorical and continuous data, respectively. Percentages do not add up to 100% due to rounding

^b^Higher scores indicate a more favorable view of biomedical research

^c^Higher scores indicate more distrust of the healthcare system

**Fig. 1:**
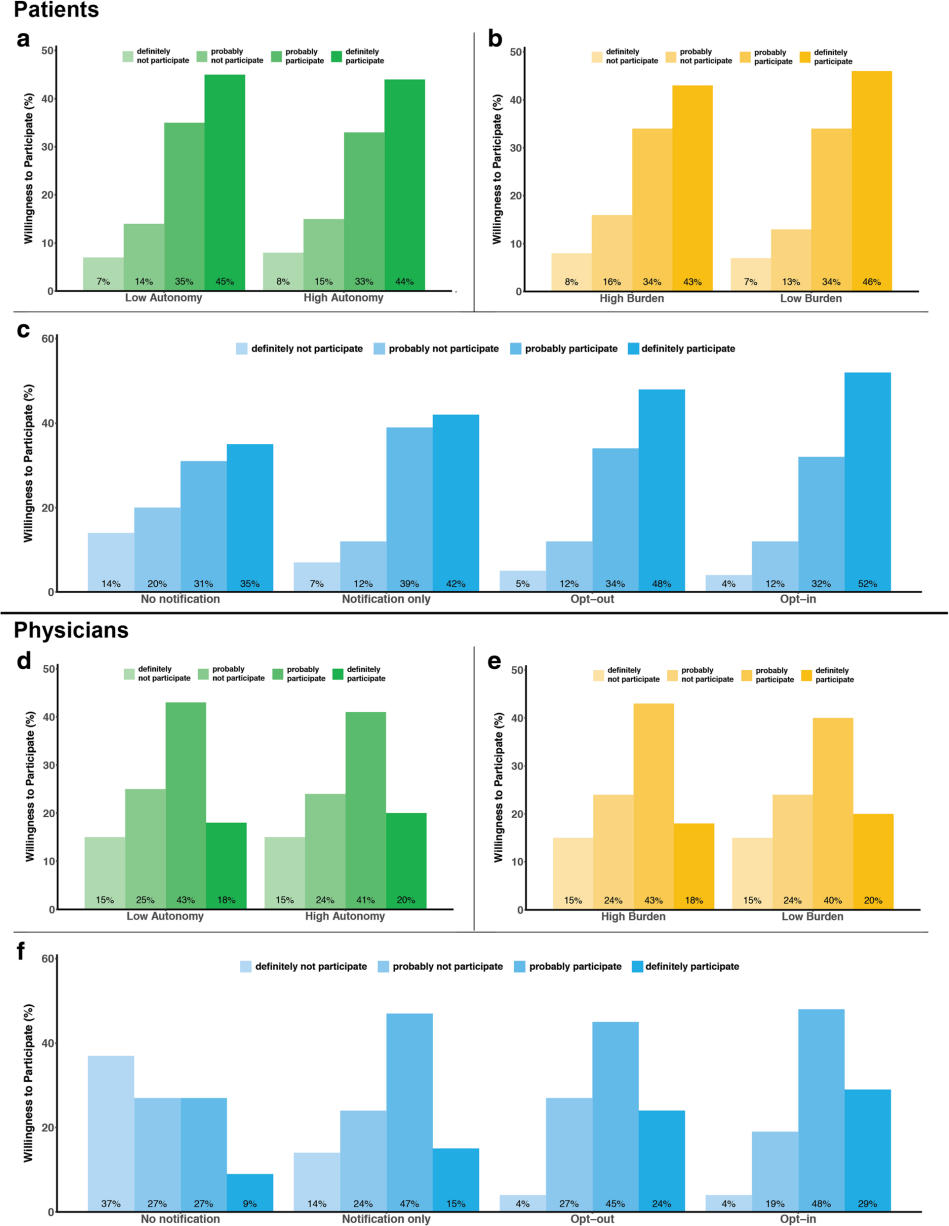
Patients’ and physicians’ willingness to participate in a pragmatic clinical trial by attribute level. Bars represent the proportion of patients (**a**-**c**) and physicians (**d**-**f**) willing to participate in a hypothetical pragmatic trial based on the level of treatment autonomy (green), research burden (yellow), and consent approach (blue)

Willingness to participate in at least one of the eight hypothetical clinical trials was high (78%). Most patients were willing to have their facility participate in the trial regardless of the level of treatment autonomy for the physician (low 79% vs. high 77%; *p* = 0.13) or research burden for the patient (low 80% vs. high 76%; *p* = 0.06). In contrast, the consent approach was influential (Table 2). More patients were willing to participate in trials that had opt-in (84%), opt-out (82%), or notification-only (81%) compared to a no-notification (66%) consent approach (*p* < 0.001 for each two-way comparison). Figure 1 displays the unadjusted results from the 4-point response scale for willingness to participate by study attribute. Associations between patient characteristics and willingness to participate are shown in Table 3. Increasing patient age was associated with increased willingness (OR 6.05 for > 70 years vs. < 40 years; 95% CI 1.64, 22.31; *p* = 0.016), and greater distrust in the healthcare system was associated with decreased willingness (OR 0.87; 95% CI 0.83, 0.92; *p* < 0.001). Only age, research attitude score, and health care system distrust score met the *p* value criterion for inclusion in the final adjusted model, along with the three trial attributes.

**Table 2:** Bivariate associations between trial attributes and patients’ willingness to have clinic participate

Attribute	Willing to participate^a^, number^b^ (percentage)	OR (95% CI)	*P* value
Autonomy			
Low	635 (79)	1.00	0.13
High	617 (77)	0.81 (0.61, 1.06)	
Burden			
Low	641 (80)	1.00	0.06
High	611 (76)	0.70 (0.48, 1.01)	
Consent			
Opt-in	336 (84)	1.00	<0.001
Opt-out	333 (82)	0.88 (0.49, 1.57)	
Notification-only	323 (81)	0.68 (0.38, 1.23)	
No-notification	260 (66)	0.19 (0.10, 0.34)	

^a^Willingness to participate was defined as the “definitely willing” or “probably willing” categories combined

^b^Denominators differ for each attribute because of the 1600 total randomized controlled trial scenarios answered (200 patients × 8 scenarios), each level of burden and autonomy was included in 50% of the scenarios (*N* = 800), whereas the four levels of consent were each included in 25% of the scenarios (*N* = 400)

**Table 3:** Bivariate associations between patient characteristics and willingness to participate

Characteristic	Willing to participate^a^, number (percentage)	OR (95% CI)	*P* value
Sex			
Male	651 (76)	1.00	0.17
Female	601 (81)	1.61 (0.81, 3.22)	
Age (years)			
<40	128 (67)	1.00	0.007
40–49	142 (71)	1.60 (0.47, 5.42)	
50–59	273 (78)	2.45 (0.83, 7.23)	
60–69	423 (83)	5.06 (1.72, 14.86)	
Over 70	286 (83)	6.48 (1.89, 22.24)	
Race			
Not black	475 (77)	1.00	0.96
Black	777 (79)	1.02 (0.50, 2.10)	
Education			
≤ High school	600 (78)	1.00	0.71
Some college	363 (81)	1.31 (0.58, 2.96)	
College or Graduate degree	289 (75)	0.89 (0.37, 2.12)	
Income			
<US$20,000	387 (82)	1.00	0.68
US$20,000–US$39,999	333 (79)	0.65 (0.27, 1.58)	
US$40,000)–US$59,999	218 (76)	0.60 (0.21, 1.72)	
Over US$60,000	294 (77)	0.61 (0.24, 1.57)	
Years on dialysis			
Less than 1 year	222 (79)	1.00	0.80
1–3 years	370 (78)	0.94 (0.33, 2.68)	
3–5 years	310 (81)	1.00 (0.36, 2.79)	
More than 5 years	350 (75)	0.66 (0.23, 1.89)	
Hospitalizations in past year			
None	397 (80)	1.00	0.80
1	222 (79)	1.03 (0.35, 2.97)	
2	243 (76)	0.69 (0.26, 1.83)	
3	390 (77)	0.73 (0.31, 1.72)	
Hypertension			
No	188 (92)	1.00	0.01
Yes	1050 (77)	0.17 (0.05, 0.68)	
Heart disease			
No	636 (77)	1.00	0.66
Yes	532 (79)	1.17 (0.58, 2.39)	
Diabetes			
No	649 (79)	1.00	0.68
Yes	519 (77)	0.86 (0.42, 1.75)	
COPD			
No	1034 (79)	1.00	0.41
Yes	134 (73)	0.61 (0.19, 1.94)	
Malignancy			
No	1064 (79)	1.00	0.54
Yes	104 (72)	0.66 (0.18, 2.46)	
Research Attitude Questionnaire^b^ (per 1-point change in score)	n/a	1.09 (1.02, 1.17)	0.01
Revised Healthcare System Distrust Scale^c^ (per 1-point change in score)	n/a	0.87 (0.83, 0.92)	<0.001

*COPD* chronic obstructive pulmonary disease

^a^Willingness to participate was defined as the “definitely willing” or “probably willing” categories combined

^b^Higher scores indicate a more favorable view of biomedical research

^c^Higher scores indicate more distrust of the health care system

When adjusted for levels of treatment autonomy and research burden, patients were less willing to participate in a clinical trial using a no-notification approach (OR 0.20; 95% CI 0.11, 0.36) compared to an opt-in approach (*p* < 0.001) (Table 4). Regardless of clinical trial attributes, age was associated with willingness to participate. For example, patients age 70 years or older were more likely to participate than those under 40 years of age (OR 6.05; 95% CI 1.64, 22.31; *p* = 0.02). Patients with greater distrust in the healthcare system were less likely to participate (OR 0.89 per 1-point increase in distrust; 95% CI 0.83, 0.95; *p* < 0.001). There was no statistically significant effect between any two attributes on willingness to participate (*p* values for all interaction terms >0.10).

**Table 4:** Influence of attributes on willingness to participate^a^ in final adjusted model

	Patient		Physician	
Characteristic	OR (95% CI)	*P* value	OR (95% CI)	*P* value
Autonomy				
Low	1.00	0.13	1.00	0.96
High	0.79 (0.59, 1.07)		1.01 (.74-1.37)	
Burden				
Low	1.00	0.06	1.00	0.79
High	0.68 (0.46, 1.01)		1.04 (.75-1.44)	
Consent				
Opt-in	1.00	<0.001	1.00	<0.001
Opt-out	0.91 (0.50, 1.63)		0.50 (0.26, 0.96)	
Notification-only	0.69 (0.38, 1.27)		0.33 (0.18, 0.59)	
No-notification	0.20 (0.11, 0.36)		0.04 (0.02, 0.08)	
Age (years)				
<40	1.00	0.02		
40–49	1.51 (0.39, 5.79)			
50–59	2.07 (0.65, 6.57)			
60–69	4.65 (1.49, 14.50)			
Over 70	6.05 (1.64, 22.31)			
Research Attitude Questionnaire^b^ (per 1-point change in score)	1.05 (0.98, 1.12)	0.20		
Revised Healthcare System Distrust Scale^c^ (per 1-point change in score)	0.89 (0.83, 0.95)	<0.001		

Although statistically significant in bivariate analyses, hypertension was not included in the final model since nearly all (91%) of the patients had hypertension

^a^Willingness to participate was defined as the “definitely willing” or “probably willing” categories combined

^b^Higher scores indicate a more favorable view of biomedical research

^c^Higher scores indicate more distrust of the healthcare system

### Physicians

Of the 1635 randomly selected physician members of the ASN who were emailed an invitation to participate, 747 (72%) opened the email and 272 (16.6%) initiated the online survey. Forty-two physicians (15.4%) were ineligible because they did not treat adults in a US outpatient dialysis unit and 27 (9.9%) did not complete the survey. Thus, 203 physicians completed the survey giving an overall response rate of 12.7%. The majority of participating physicians were male, white, 5 or more years beyond fellowship, and self-identified as having experience as a clinical trial investigator (Additional file 1: Appendix C). Demographics of responders were compared with those of a random sample of 300 non-responders. There were no significant differences in either the gender (*p* = 0.68) or mean age (*p* = 0.34) of responding and non-responding physicians.

Similar to the patient participants, most physician participants were willing to have their dialysis facility participate in at least one of the eight hypothetical clinical trials (61%). Physicians’ willingness to participate was not affected by either treatment autonomy (61% vs. 61% for low and high, respectively; *p* = 0.96) or research burden (60% vs. 61% for low and high, respectively; *p* = 0.79). Physicians were generally less willing than patients to have dialysis facilities participate in the described trials across all scenarios (Fig. 1), and the effect of consent approach on willingness to participate appeared to be stronger for physicians than for patients (Table 4). However, statistical comparisons between patients and physicians were not performed, as these were not specified in our analytic plan. As with patients, interactions among the three trial attributes were not evident among physicians (*p* > 0.1 for all interaction terms). Additionally, none of the physician characteristics was associated with willingness to participate (Additional file 1: Appendix D).

## Discussion

In this study of patients with dialysis-dependent end-stage renal disease and physicians, we did not detect an effect of curtailed treatment autonomy or research burden on willingness to participate in a hypothetical pragmatic trial, but did find an effect of the approach to consent. Both patients and physicians indicated levels of comfort that were similar for opt-out, participant notification, and traditional opt-in consent, but were less willing to participate when participants would receive no notification about the trial. However, even with no notification, two thirds of patients indicated willingness to have their facilities participate.

Patients and physicians are accustomed to interactions in which decisions about treatment approaches are either shared between the patient and the physician or directed by the physician. In traditional RCTs, therapeutic approaches are determined by chance rather than by choice [34], and such restrictions may dissuade patients or physicians from participating in the study. However, in cluster-randomized pragmatic trials patients may not have an option to avoid trials that restrict treatment autonomy because entire hospitals or clinics may be assigned to provide the same treatment approach for all eligible patients. Even when randomization is at the individual patient level, for some pragmatic trials the requirement for informed consent is waived and thus access to alternative treatments is eliminated. Interestingly, for patients and physicians in the current study, limitations on treatment autonomy did not reduce willingness to have their dialysis facility participate in the trial. Moreover, patients were as likely to participate in a trial that relied on opt-out consent or a notification of enrollment as in a trial that used opt-in informed consent, and two thirds of patients were willing to participate even in the absence of any notification. This finding is particularly notable in light of increased distrust in medical care and biomedical research among African Americans [35], who constituted the majority of participants in this ESRD cohort. Indeed, we found that race was not associated with willingness to participate in the hypothetical pragmatic trials.

Strikingly, the hypothetical pragmatic trials presented in our surveys were more acceptable to patients than physicians. This may be explained, at least in part, by the desire of some physicians to rely on their own experience to determine the best treatment for an individual patient [24]. However, notably, one third of physicians expressed willingness to have their dialysis facility participate in a trial conducted without any patient notification. Another interesting finding is that, like patients, physicians’ willingness to participate was not influenced by degree of burden to patients resulting from the trial. The absence of an effect of this attribute might be due to the small incremental burdens for this group of patients of extra testing and blood collection relative to thrice-weekly hemodialysis treatments. It is also possible that effects smaller than we were powered to detect might be evident in a larger sample.

Overall, our findings suggest that a pragmatic clinical trial comparing standard-of-care interventions for blood pressure management is generally acceptable to patients receiving maintenance dialysis and to their treating physicians. Among those we evaluated, the only influential attribute for patients and physicians was the consent approach, a finding that is relevant to ongoing discussions about the use of alternatives to the “traditional” opt-in consent in pragmatic clinical trials [36]. In particular, so long as studies were transparent and included notification to patients, they were generally acceptable to both patients and physicians. Understanding the views of multiple stakeholders is important for informing these discussions. A strength of this study is that we assessed the views of both patents and physicians, and found generally similar results for these two groups.

The findings of our study need to be considered in light of several limitations. The most important is that responses of the patients and physicians to hypothetical RCTs may not reflect the decisions they would make if they were considering participation in actual trials. However, structured vignettes have been shown to be a valid methodology in other clinical settings for producing results that accurately reflect those from more expensive and time-intensive non-hypothetical experimental procedures [37], and hypothetical preferences are the single best predictor of future enrollment decisions [38]. A second limitation is that the valuations of these trial attributes and willingness to participate may not reflect those of patients receiving hemodialysis in other geographic areas or with other diseases, or physicians from other specialties. Likewise, results may vary among trials testing different types of interventions. We chose to focus on hemodialysis patients and physicians because of the potential for dialysis care delivery as a setting for pragmatic trials [39]. The findings from this study should inform future studies in different clinical contexts. An additional limitation is the low survey response rate by physicians that occurred despite pilot testing the study instrument and offering a US$50 financial incentive conditional on completing the survey. Physicians are historically difficult to engage in survey research [40], and we acknowledge the possibility of non-response bias in the physician component of this study. Somewhat reassuringly, the age and sex of our respondents mirrored those of a random sample of physician members of the ASN. Finally, the hypothetical trials used in this study tested only three trial attributes, and it is possible that additional attributes (e.g., risk to patients) may influence participation decisions. However, we informed our attribute selection from interviews with patients and nephrologists [24], and chose to constrain the number of attributes to avoid overburdening participants.

## Conclusion

This study provides evidence that limitations placed on treatment autonomy in clinical trials may not be an important contributor to patients’ or physicians’ willingness to participate, and that patients may place similar value on a variety of consent approaches as long as there is some form of notification. These findings contribute important stakeholder perspectives to the ongoing debate about the applicability of traditional research regulations to studies involving standard-of-care interventions [4, 16, 17, 21, 22, 36, 41–43]. Future work should aim to elucidate factors driving the expressed attribute valuations and expand this evidence base to other clinical contexts.

## Additional file

Additional file 1: Supplemental study results including patient and physician surveys (A); patient scores on the pre-test research knowledge questionnaire (B); physician characteristics (C); and associations with physicians’ willingness to participate (D). (DOCX 6242 kb)

## References

[1] Ford I, Norrie J (2016). Pragmatic Trials. N Engl J Med..

[2] Institute of Medicine (2007). The learning health care system workshop summary.

[3] Institute of Medicine (2012). Best care at lower cost: the path to continuously learning health care in America.

[4] Anderson ML, Califf RM, Sugarman J (2015). Ethical and regulatory issues of pragmatic cluster randomized trials in contemporary health systems. Clin Trials..

[5] Emanuel EJ, Schnipper LE, Kamin DY, Levinson J, Lichter AS (2003). The costs of conducting clinical research. J Clin Oncol..

[6] Nathan RA (1999). How important is patient recruitment in performing clinical trials?. J Asthma..

[7] Hunningshake DB, Darby CA, Probstfield JL (1987). Recruitment experience in clinical trials: Literature summary and annotated bibliography. Control Clin Trials..

[8] Meinert CL (1986). Clinical trials: Design, conduct, and analysis.

[9] Probstfield JL, Wittes JT, Hunningshake DB (1987). Recruitment in the NHLBI population-based studies and randomized clinical trials: data analysis and survey results. Control Clin Trials..

[10] Kramer MS, Shapiro SH (1984). Scientific challenges in the application of randomized trials. JAMA..

[11] Vollmer VM, Osborn ML, Hertert S (1994). Recruiting hard-to-reach subjects: Is it worth the effort?. Control Clin Trials..

[12] Halpern SD (2002). Prospective preference assessment: a method to enhance the ethics and efficiency of randomized controlled trials. Control Clin Trials..

[13] Lovato LC, Hill K, Hertert S, Hunningshake DB, Probstfield JL (1997). Recruitment for controlled clinical trials: literature summary and annotated bibliography. Control Clin Trials..

[14] Ross S, Grant A, Counsell C, Gillespie W, Russell I, Prescott R (1999). Barriers to participation in randomised controlled trials: a systematic review. J Clin Epidemiol..

[15] Taylor KM, Feldstein ML, Skeel RT, Pandya KJ, Ng P, Carbone PP (1994). Fundamental dilemmas of the randomized clinical trial process: results of a survey of the 1,737 Eastern Cooperative Oncology Group investigators. J Clin Oncol..

[16] Faden RR, Kass NE, Goodman SN, Pronovost P, Tunis S, Beauchamp TL (2013). An ethics framework for a learning health care system: a departure from traditional research ethics and clinical ethics. Hastings Cent Rep.

[17] Solomon MZ, Bonham AC (2013). Ethical oversight of research on patient care. Hastings Cent Rep..

[18] Platt R, Kass NE, McGraw D (2014). Ethics, regulation, and comparative effectiveness research: time for a change. JAMA..

[19] Faden RR, Beauchamp TL, Kass NE (2014). Informed consent, comparative effectiveness, and learning health care. N Engl J Med..

[20] Kim SY, Miller FG (2016). Waivers and alterations to consent in pragmatic clinical trials: respecting the principle of respect for persons. IRB..

[21] Kass NE, Faden RR, Goodman SN, Pronovost P, Tunis S, Beauchamp TL (2013). The Research-treatment distinction: a problematic approach for determining which activities should have ethical oversight. Hastings Cent Rep..

[22] Largent EA, Joffe S, Miller FG (2011). Can research and care be ethically integrated?. Hastings Cent Rep..

[23] Sugarman J (2016). Ethics of research in usual care settings: data on point. AJOB Empir Bioeth..

[24] Kraybill A, Dember LM, Joffe S (2016). Patient and physician views about protocolized dialysis treatment in randomized trials and clinical care. AJOB Empir Bioeth..

[25] Bridges JF, Hauber AB, Marshall D (2011). Conjoint analysis applications in health–a checklist: a report of the ISPOR Good Research Practices for Conjoint Analysis Task Force. Value Health..

[26] Rubright JD, Cary MS, Karlawish J, Kim SYH (2011). Measuring how people view biomedical research: reliability and validity analysis of the Research Attitudes Questionnaire. J Empir Res Hum Res Ethics..

[27] Shea JA, Micco E, Dean LT, McMurphy S, Schwartz JS, Armstrong K (2008). Development of a revised Health Care System Distrust scale. J Gen Intern Med..

[28] Williams RL (2000). A note on robust variance estimation for cluster-correlated data. Biometrics..

[29] Rogers WH. Stata Tehcnical Bulletin: regression standard errors in clustered samples. 1993;3(13):19-23. Available at http://stata-press.com/journals/stbcontents/stb13.pdf.

[30] Localio AR, Berlin JA, Ten Have TR, Kimmel SE (2001). Adjustments for center in multicenter studies: an overview. Ann Intern Med..

[31] Kerry SM, Bland JM (1998). The intracluster correlation coefficient in cluster randomisation. BMJ..

[32] 2015 End Stage Renal Disease (ESRD) Network 4 Annual Report. November 30, 2016. (Accessed September 13, 2017, at https://www.qirn4.org/Files/Data-CrownWeb/Annual-Reports/2015_Network_Annual_Report-Network4.aspx.).

[33] Armstrong K, McMurphy S, Dean LT (2008). Differences in the patterns of health care system distrust between blacks and whites. J Gen Intern Med..

[34] Marquis D (1983). Leaving therapy to chance. Hastings Cent Rep..

[35] Corbie-Smith G, Thomas SB, St George DM (2002). Distrust, race, and research. Arch Intern Med..

[36] McKinney RE, Beskow LM, Ford DE (2015). Use of altered informed consent in pragmatic clinical research. Clin Trials..

[37] Peabody JW, Luck J, Glassman P, Dresselhaus TR, Lee M (2000). Comparison of vignettes, standardized patients, and chart abstraction: a prospective validation study of 3 methods for measuring quality. JAMA..

[38] Halpern SD, Metzger DS, Berlin JA, Ubel PA (2001). Who will enroll? Predicting participation in a phase II AIDS vaccine trial. J Acq Immun Def Synd..

[39] Dember LM, Archdeacon P, Krishnan M (2016). Pragmatic Trials in Maintenance Dialysis: Perspectives from the Kidney Health Initiative. J Am Soc Nephrol..

[40] Keating NL, Zaslavsky AM, Goldstein J, West DW, Ayanian JZ (2008). Randomized trial of $20 versus $50 incentives to increase physician survey response rates. Med Care..

[41] Sugarman J, Califf RM (2014). Ethics and regulatory complexities for pragmatic clinical trials. JAMA..

[42] Kim SY, Miller FG (2014). Informed consent for pragmatic trials–the integrated consent model. N Engl J Med..

[43] Chen SC, Kim SY (2016). A framework for analysis of research risks and benefits to participants in standard of care pragmatic clinical trials. Clin Trials..

