# Supplementary material for: Willingness to participate in pragmatic dialysis trials: the importance of physician decisional autonomy and consent approach
Source: Trials. 2017 Oct 11;18:474. doi: 10.1186/s13063-017-2217-8 (PMC5637128; doi:10.1186/s13063-017-2217-8)
Supplement: Additional file 1: — Supplemental study results including patient and physician surveys (A); patient scores on the pre-test research knowledge questionnaire (B); physician characteristics (C); and associations with physicians’ willingness to participate (D). (DOCX 6242 kb) [file 13063_2017_2217_MOESM1_ESM.docx]

**ADDITIONAL FILE 1**

**Willingness to Participate in Pragmatic Dialysis Trials: The Importance of Physician Decisional Autonomy and Consent Approach**

Katherine R. Courtright, MD MS

Scott D. Halpern, MD PhD MBE

Steven Joffe, MD MPH

Susan S. Ellenberg, PhD

Jason Karlawish, MD

Vanessa Madden, BS

Nicole B. Gabler, PhD MHA

Stephanie Szymanski, BA

Kuldeep Yadav, BA

Laura M. Dember, MD

Appendix A. Patient and physician surveys

Appendix B. Patient scores on the pre-test research knowledge questionnaire

Appendix C. Physician characteristics

Appendix D. Associations with physicians’ willingness to participate

**Appendix A. Patient and physician surveys**

|  | **PATIENT SURVEY** | **PHYSICIAN SURVEY** |
| --- | --- | --- |
|  | **PART 1: WHAT IS RESEARCH?**  First, let’s learn about research studies (cartoon, video).  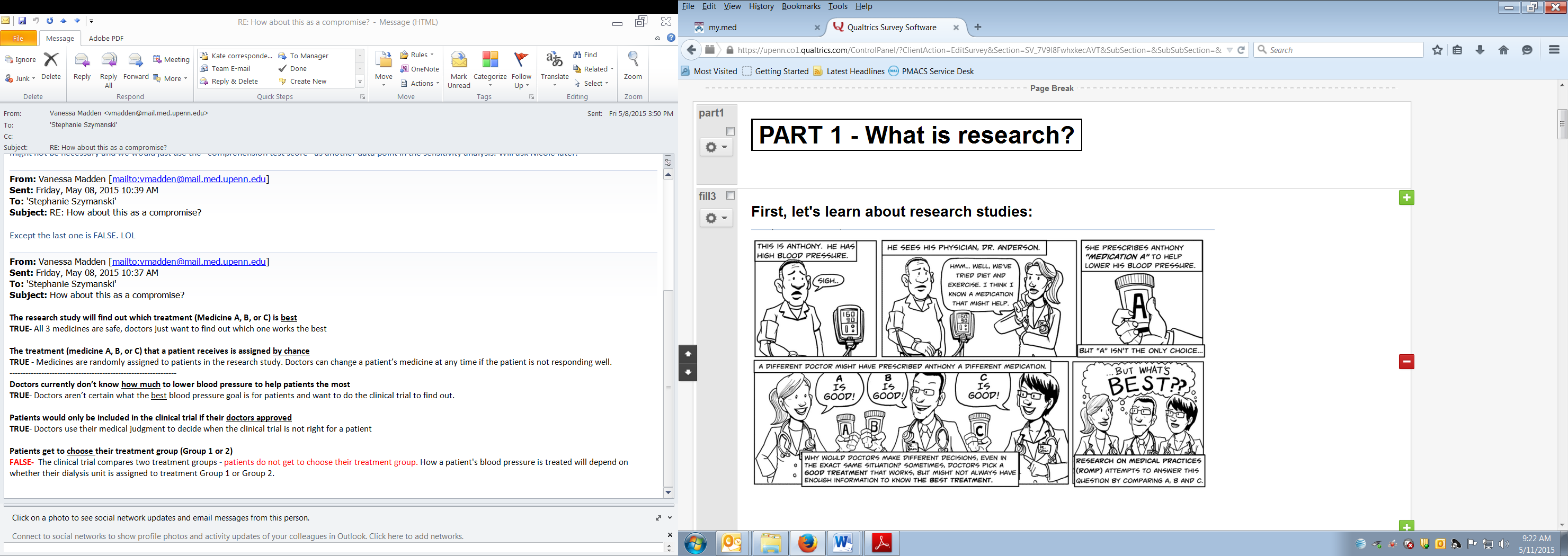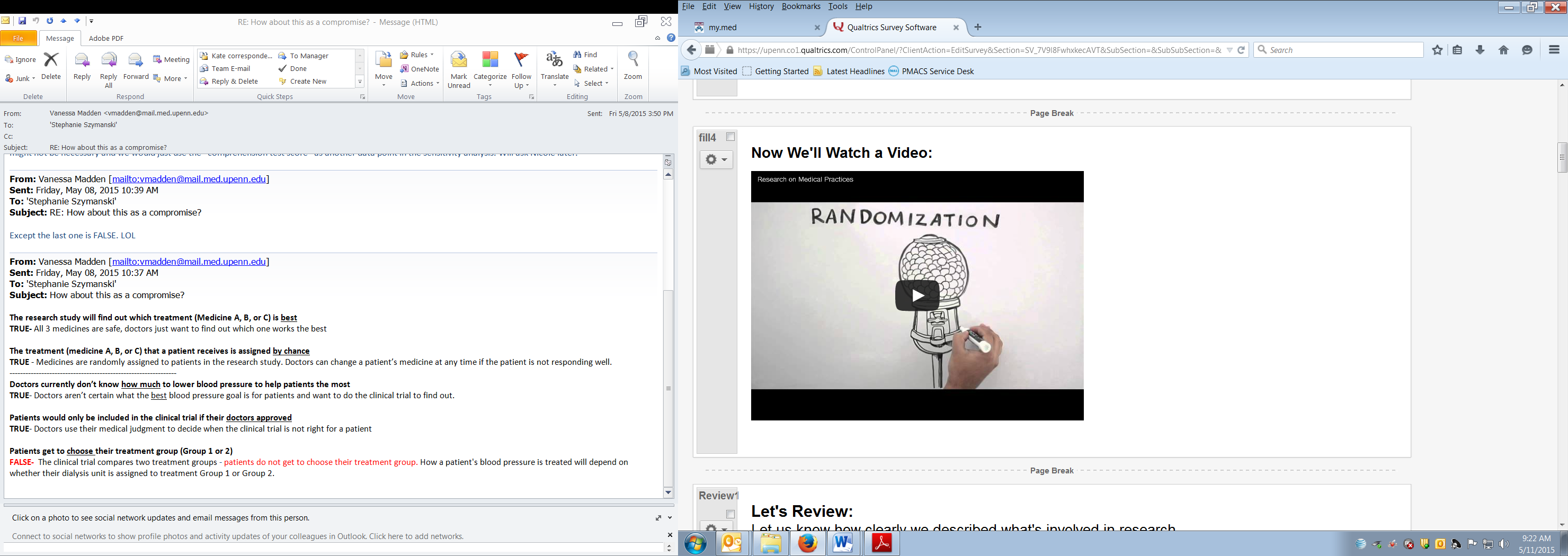  **LET’S REVIEW:**  Let us know how clearly we described what's involved in research, by answering these 2 questions:   - **The purpose of this research study is to find out which treatment (Medicine A, B, or C) is best** [**TRUE-** All 3 medicines are safe, doctors just want to find out which one works the best] - **The treatment (Medicine A, B, or C) that a patient receives is assigned by chance [TRUE** - Medicines are randomly assigned to patients in the research study. Doctors can change a patient’s medicine at any time if the patient is not responding well.] |  |
|  | **PART 2: WHAT WILL I BE ASKED TO DO?**  You will now be shown 8 imaginary research studies. These studies are about blood pressure control for patients with kidney disease.  We want to know how you feel about different features of research studies.  **BACKGROUND**  Many patients with kidney disease have high blood pressure. High blood pressure raises risks for other medical problems, such as heart attacks and strokes. Dialysis helps to clean the blood and control blood pressure, but doctors may also use medicines to help control patients’ blood pressure. However, currently doctors do not know how much to lower the blood pressure to help patients the most.  **LET’S IMAGINE**  Imagine that doctors across the U.S. are doing a research study to learn about how much to lower blood pressure. The goal of this study is to help doctors provide better care for dialysis patients in the future. | **WHAT WILL I BE ASKED TO DO?**  On the next screens you will be presented with a series of hypothetical clinical trials, all focused on management of hypertension in hemodialysis patients.  We want to understand how different features of randomized trials might influence your decision to have your dialysis facility participate in a clinical trial.  **HYPOTHETICAL CLINICAL TRIAL DESIGN**  Despite the prevalence of hypertension among hemodialysis patients, the optimal BP target is currently not known. Please imagine that investigators are conducting a large, adequately powered clinical trial to evaluate the effect of BP targets and management strategies on clinical outcomes.  In order to understand your views about clinical trials you will be:   - presented with 8 hypothetical clinical trials, and - asked about your willingness to have your dialysis facility, and thus, your patients participate in each hypothetical trial. |
|  | **HOW WOULD THE IMAGINARY STUDY WORK?**  The research study - called a clinical trial - would compare 2 groups of patients. All patients in Group 1 would have their blood pressure managed in one way, and all patients in Group 2 would have their blood pressure managed a different way.  Patients would not be included in the study if their doctor thought they should not participate.  **WHICH GROUP COULD A PATIENT BE IN?**  Patients do not choose which treatment approach they get. Instead, which group they are in is assigned as part of the study.  Groups are “randomized” by dialysis unit  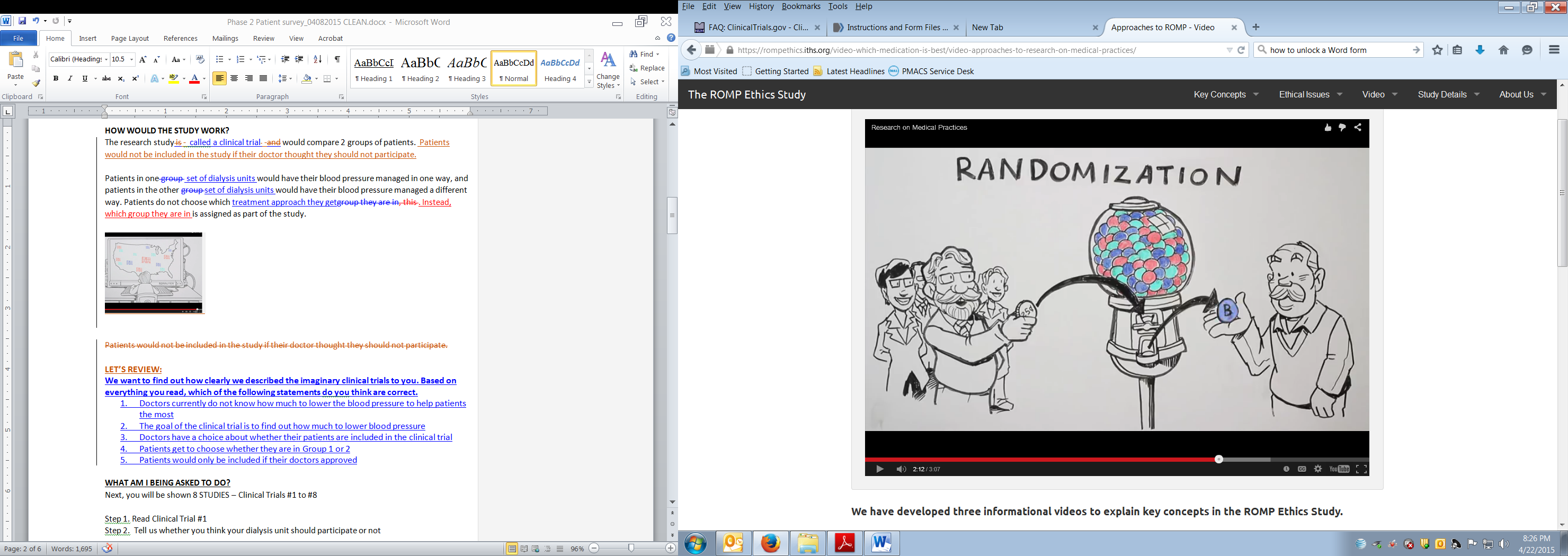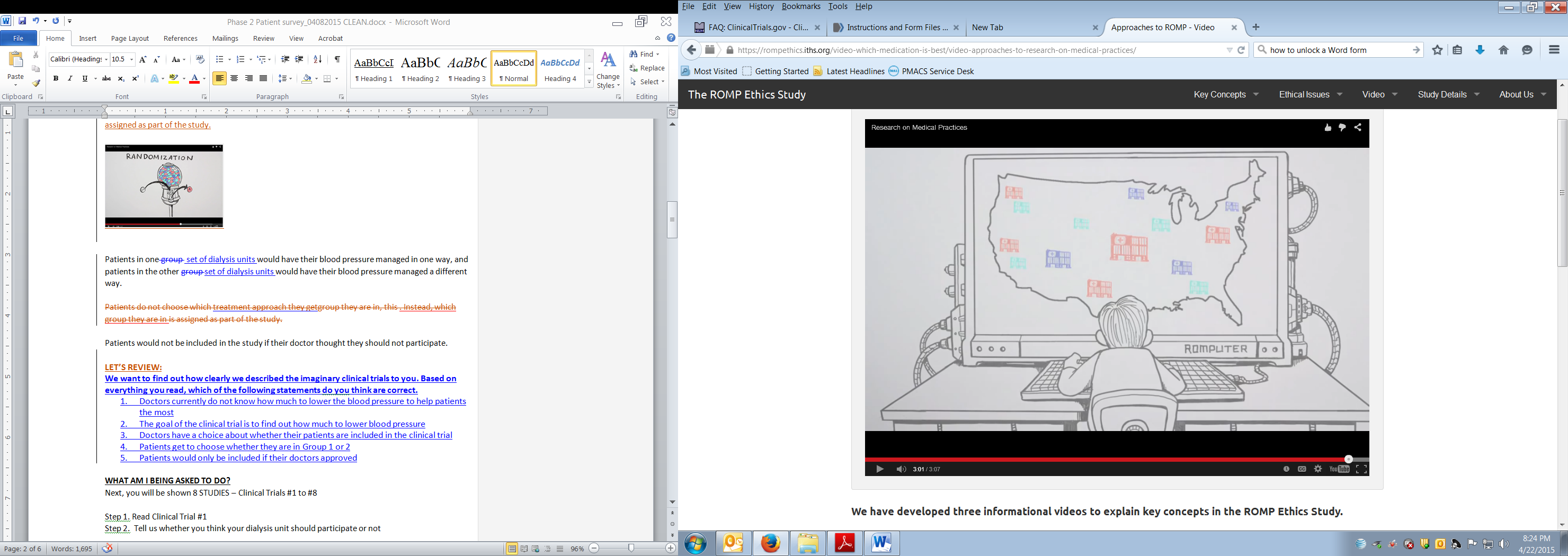  This means that the treatment approach a patient receives depends on which dialysis unit they go to. | All 8 trials have the following in common:  **1) Unit of Randomization:** Dialysis facilities, rather than individual patients, are randomized to the treatment group  **2) Treatment Assignment:** Dialysis facilities are randomly assigned to one of the two possible treatment groups **3) Outcomes: The same clinical outcomes will be assessed in all trials.**  **4) Trial duration:** 1 year for all dialysis facilities **5) Oversight:**  Provided by Institutional Review Boards and a Data and Safety Monitoring Board |
|  | **LET’S REVIEW:**  Let us know how clearly we described the clinical trial, by answering these 3 questions:   - **Doctors currently don’t know how much to lower blood pressure to help patients the most [TRUE**- Doctors aren’t certain what the best blood pressure goal is for patients and want to do the clinical trial to find out.] - **Patients would only be included in the clinical trial if their doctors approved [TRUE**- Doctors use their medical judgment to decide when the clinical trial is not right for a patient] - **Patients get to choose their treatment group (Group 1 or 2) [FALSE-** The clinical trial compares two treatment groups - patients do not get to choose their treatment group. How a patient's blood pressure is treated will depend on whether their dialysis unit is assigned to treatment Group 1 or Group 2.] |  |
|  | **WHAT AM I BEING ASKED TO DO?**  Next, you will be shown 8 clinical trials  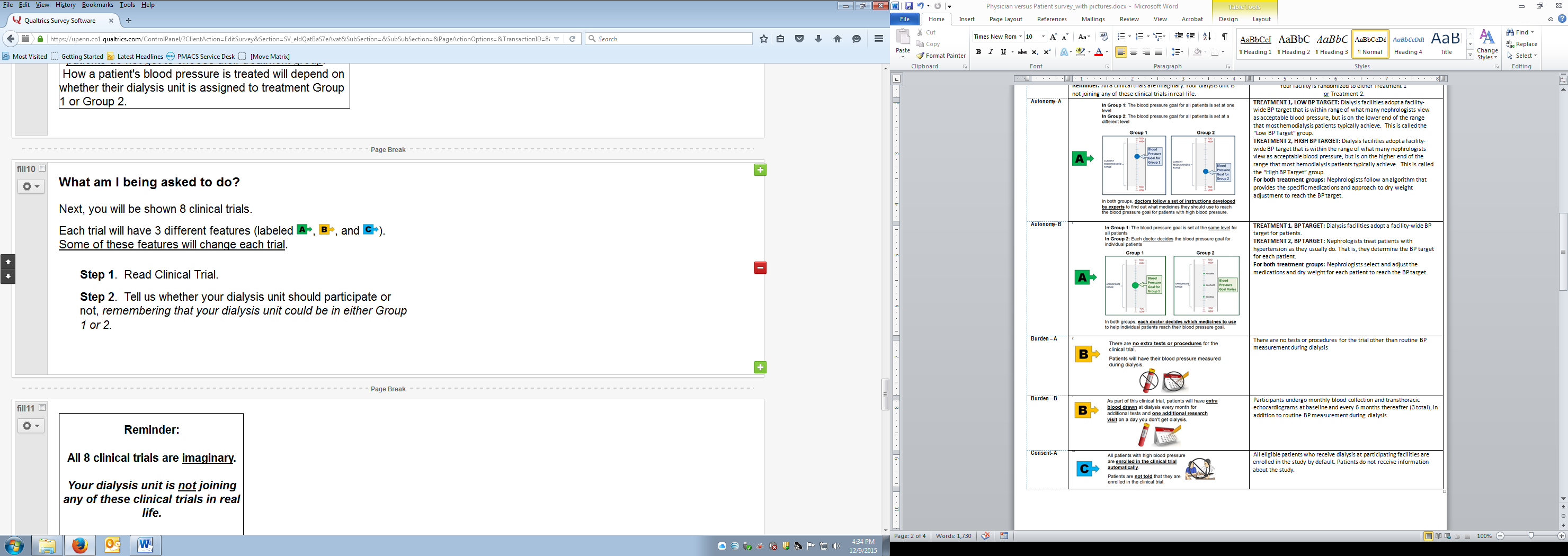.  Some of these features will change each trial.   - Step 1. Read Clinical Trial - Step 2. Tell us whether you think your dialysis unit should participate or not, remembering that your dialysis unit could be in either Group 1 or Group 2   **Reminder:** All 8 clinical trials are imaginary. Your dialysis unit is not joining any of these clinical trials in real-life. | **CLICK TO VIEW HYPOTHETICAL CLINICAL TRIAL #1**  **Reminder:** We are interested in your perspectives on various features of clinical trials, and are using hypertension management as an example.  After the trial description, you will be asked about your willingness to have your hemodialysis facility, and thus, your patients participate in the hypothetical clinical trial.  **[Randomization schematic]**  Your facility is randomized to either Treatment 1 or Treatment 2. |
| **Autonomy- A** | 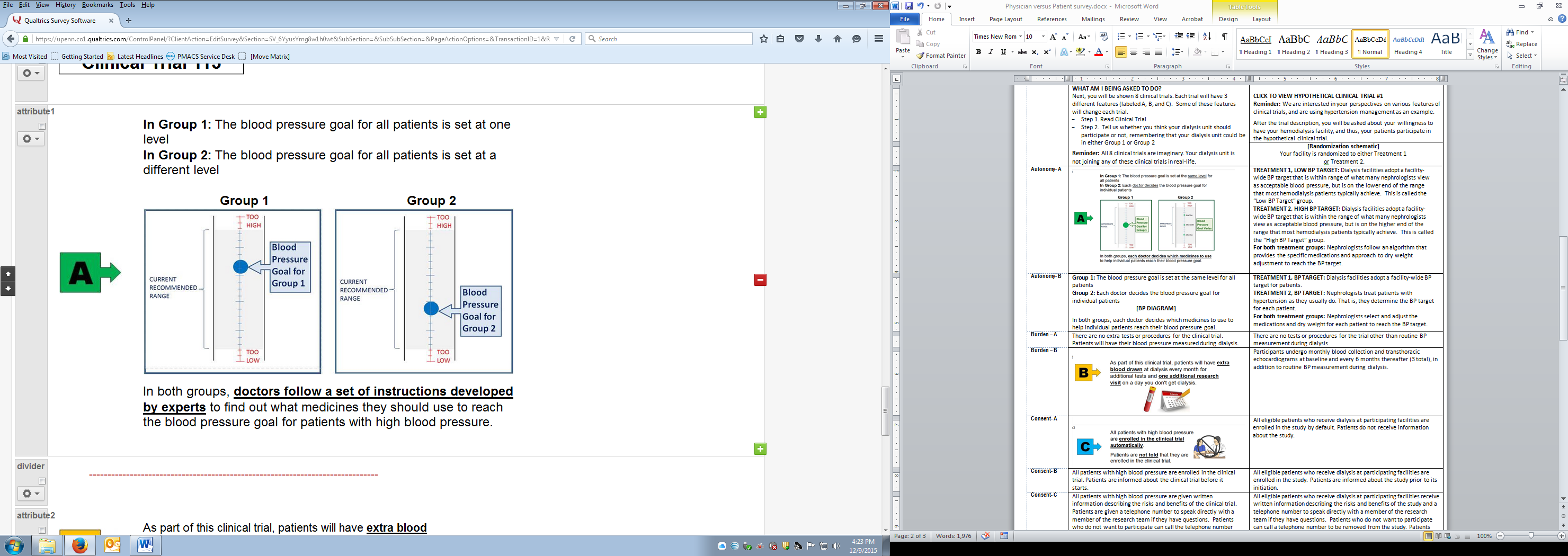 | **TREATMENT 1, LOW BP TARGET:** Dialysis facilities adopt a facility-wide BP target that is within range of what many nephrologists view as acceptable blood pressure, but is on the lower end of the range that most hemodialysis patients typically achieve. This is called the “Low BP Target” group.  **TREATMENT 2, HIGH BP TARGET:** Dialysis facilities adopt a facility-wide BP target that is within the range of what many nephrologists view as acceptable blood pressure, but is on the higher end of the range that most hemodialysis patients typically achieve. This is called the “High BP Target” group.  **For both treatment groups:** Nephrologists follow an algorithm that provides the specific medications and approach to dry weight adjustment to reach the BP target. |
| **Autonomy- B** | 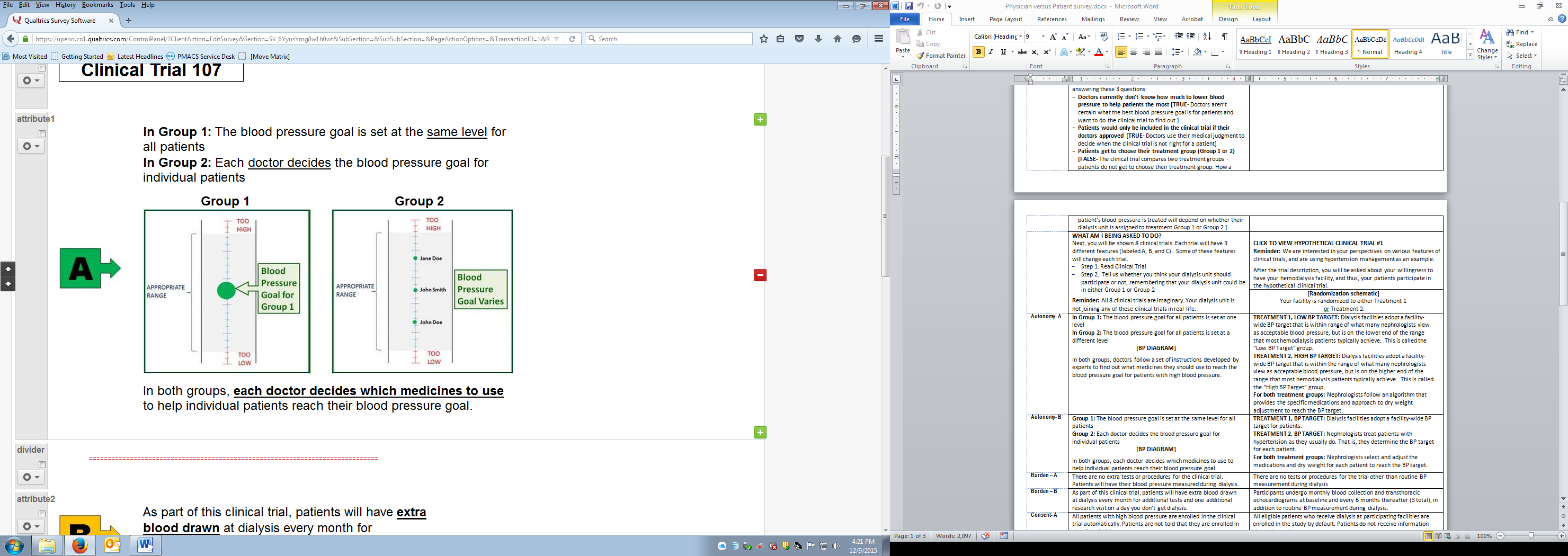 | **TREATMENT 1, BP TARGET:** Dialysis facilities adopt a facility-wide BP target for patients.  **TREATMENT 2, BP TARGET:** Nephrologists treat patients with hypertension as they usually do. That is, they determine the BP target for each patient.  **For both treatment groups:** Nephrologists select and adjust the medications and dry weight for each patient to reach the BP target. |
| **Burden – A** | 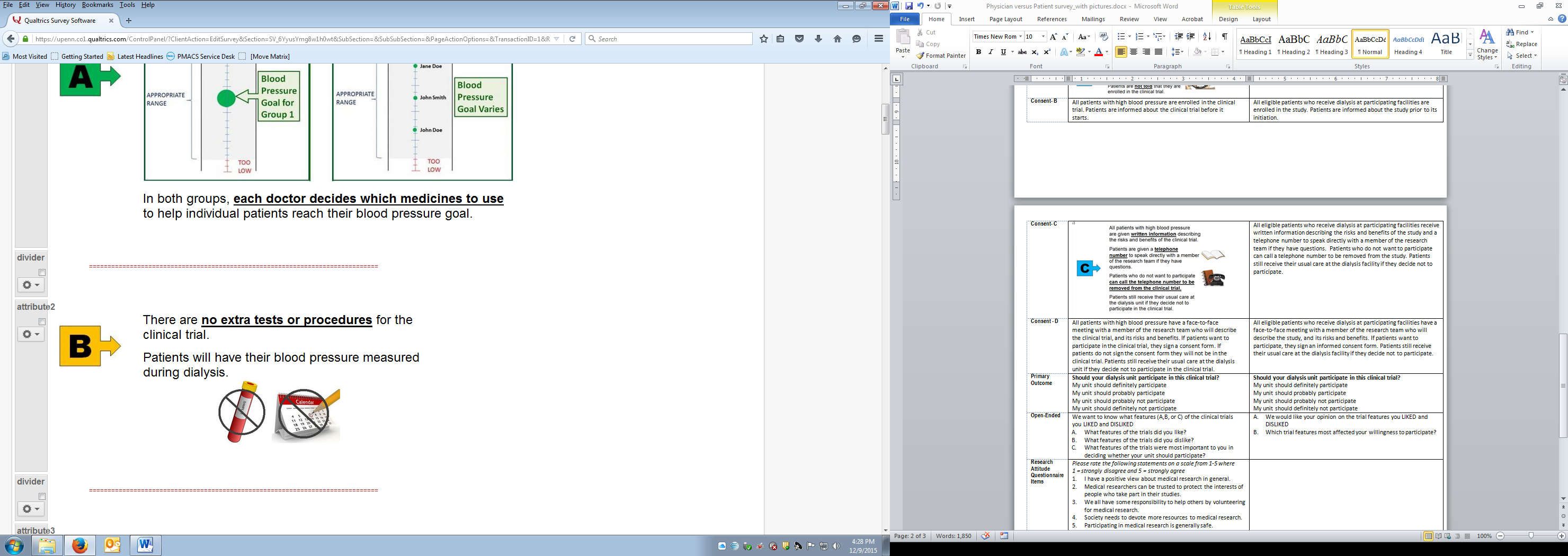 | There are no tests or procedures for the trial other than routine BP measurement during dialysis |
| **Burden – B** | 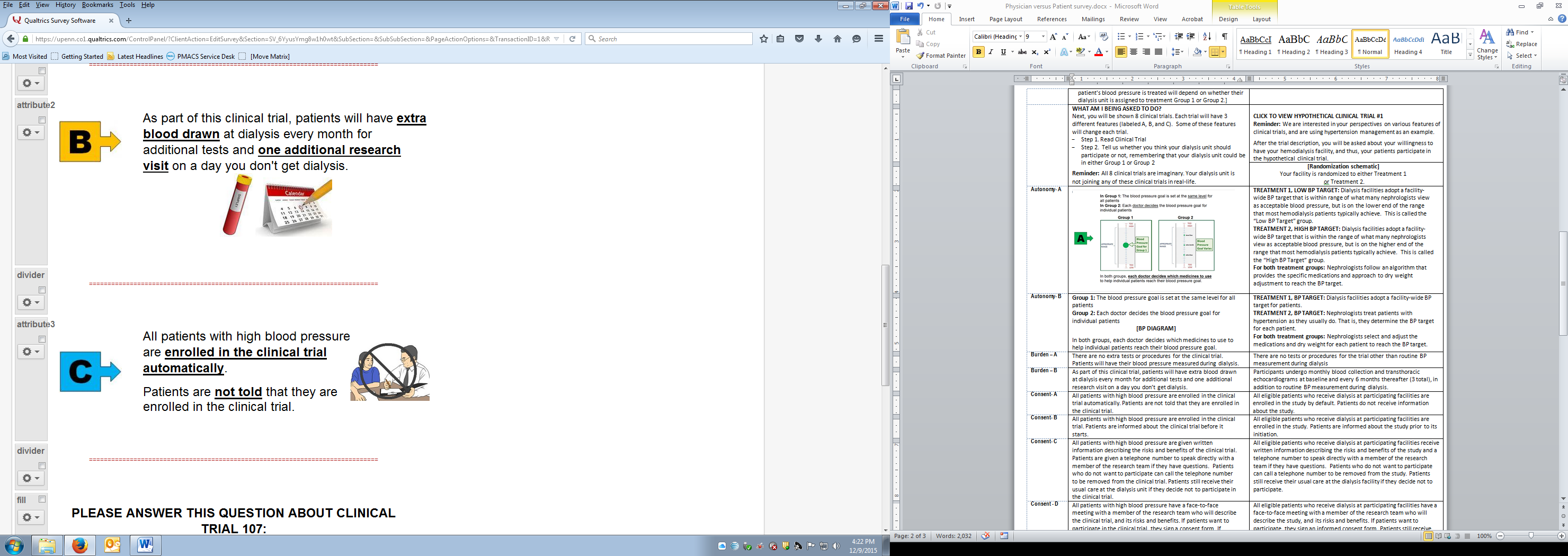 | Participants undergo monthly blood collection and transthoracic echocardiograms at baseline and every 6 months thereafter (3 total), in addition to routine BP measurement during dialysis. |
| **Consent- A** | 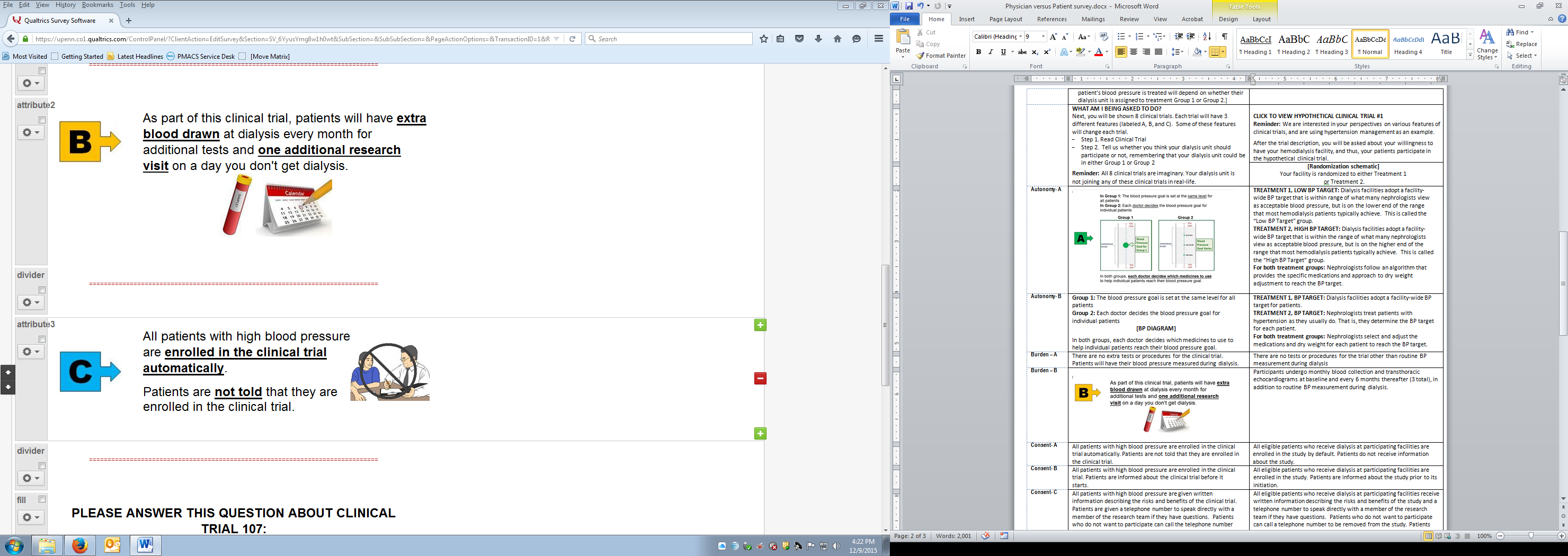 | All eligible patients who receive dialysis at participating facilities are enrolled in the study by default. Patients do not receive information about the study. |
| **Consent- B** | 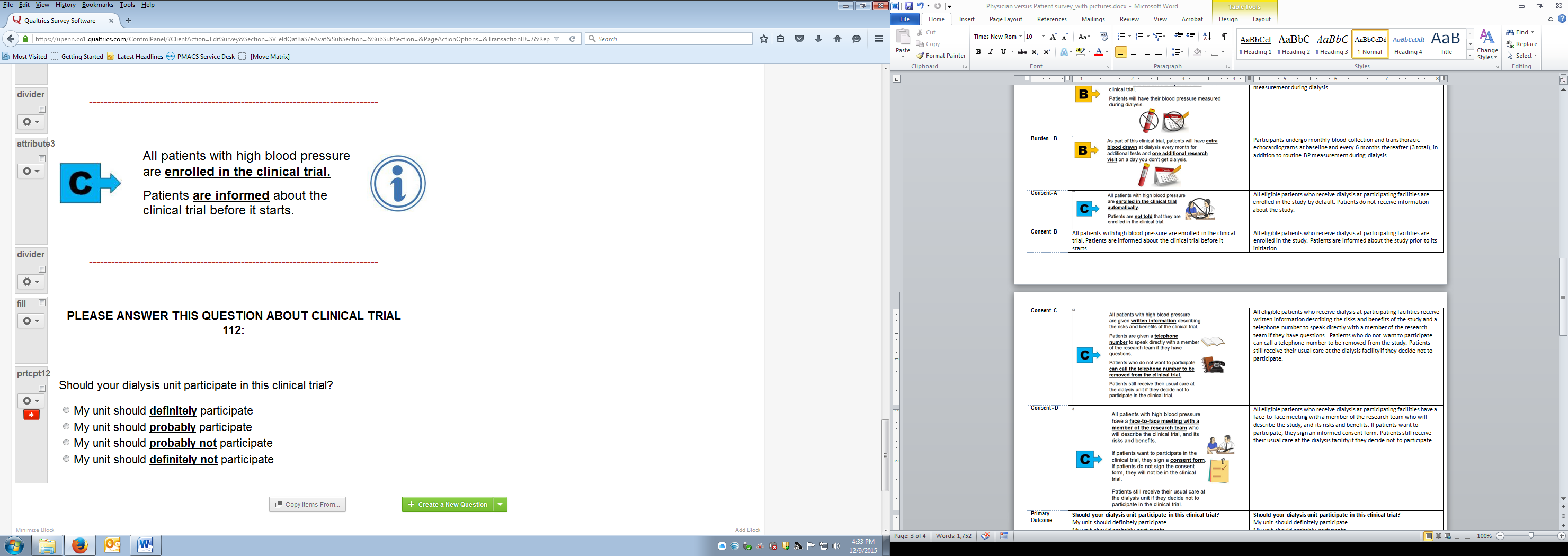 | All eligible patients who receive dialysis at participating facilities are enrolled in the study. Patients are informed about the study prior to its initiation. |
| **Consent- C** | 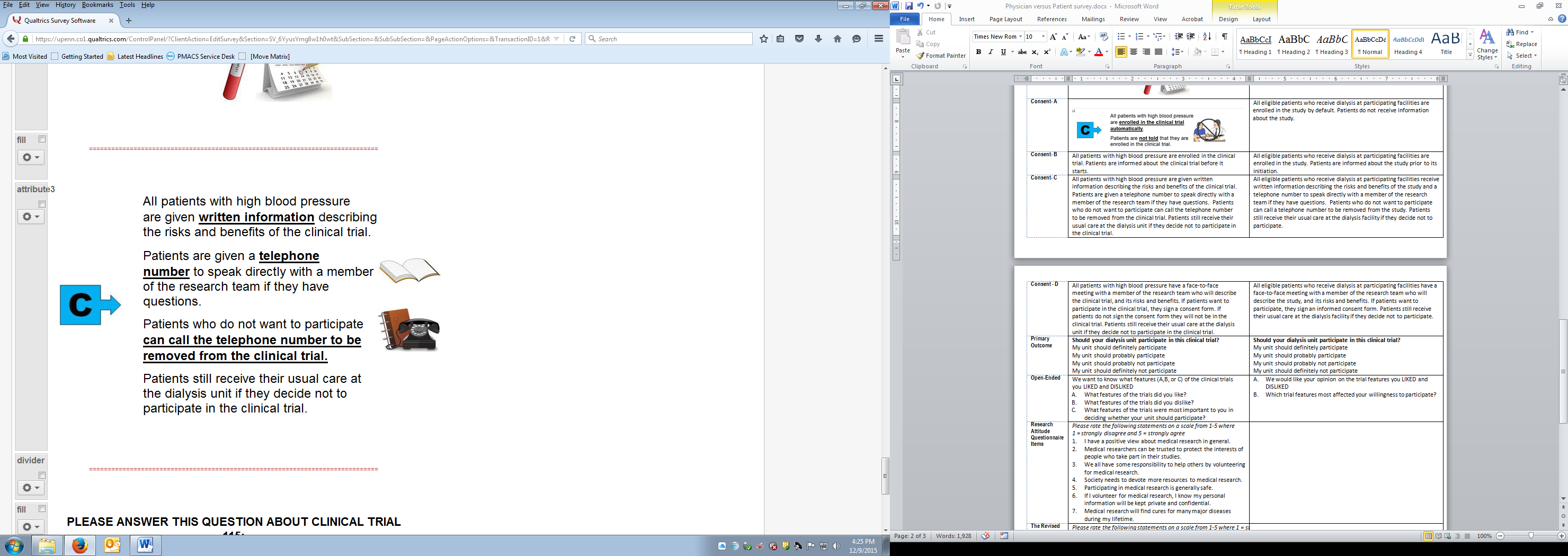 | All eligible patients who receive dialysis at participating facilities receive written information describing the risks and benefits of the study and a telephone number to speak directly with a member of the research team if they have questions. Patients who do not want to participate can call a telephone number to be removed from the study. Patients still receive their usual care at the dialysis facility if they decide not to participate. |
| **Consent - D** | 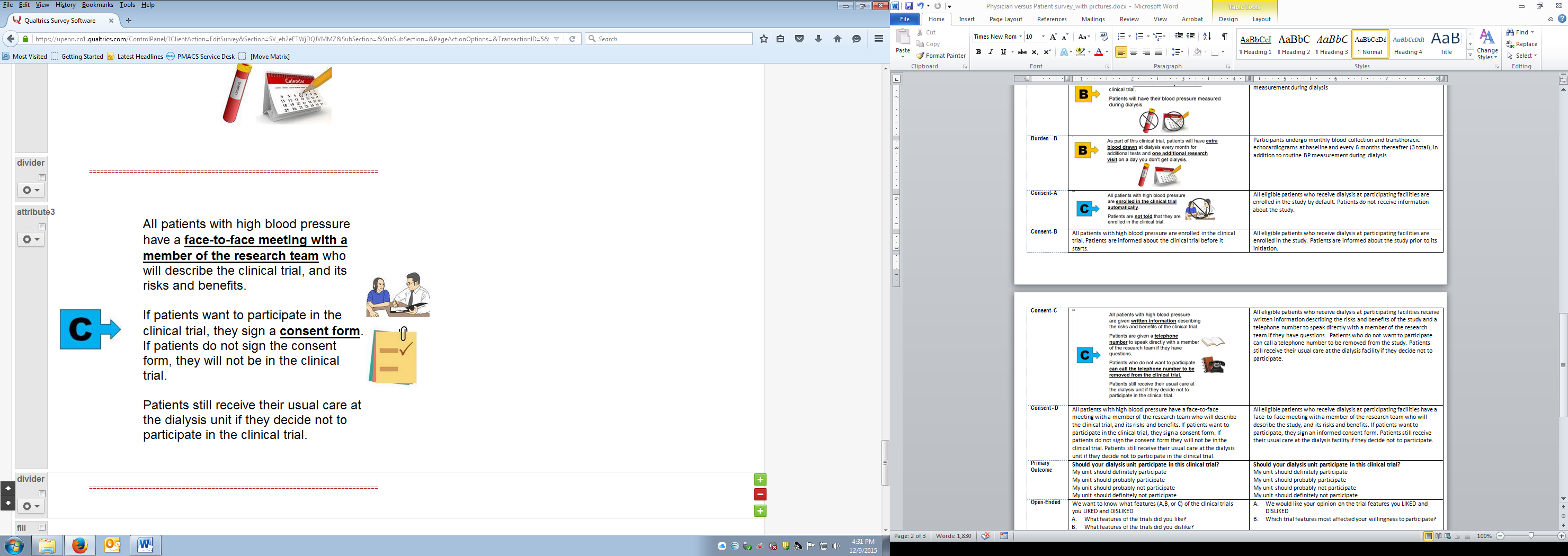 | All eligible patients who receive dialysis at participating facilities have a face-to-face meeting with a member of the research team who will describe the study, and its risks and benefits. If patients want to participate, they sign an informed consent form. Patients still receive their usual care at the dialysis facility if they decide not to participate. |
| **Primary Outcome** | **Should your dialysis unit participate in this clinical trial?**  My unit should definitely participate  My unit should probably participate  My unit should probably not participate  My unit should definitely not participate | **Should your dialysis unit participate in this clinical trial?**  My unit should definitely participate  My unit should probably participate  My unit should probably not participate  My unit should definitely not participate |
| **Open-Ended** | We want to know what features (A,B, or C) of the clinical trials you LIKED and DISLIKED   1. What features of the trials did you like? 2. What features of the trials did you dislike? 3. What features of the trials were most important to you in deciding whether your unit should participate? | 1. We would like your opinion on the trial features you LIKED and DISLIKED 2. Which trial features most affected your willingness to participate? |
| **Research Attitude Questionnaire Items** | *Please rate the following statements on a scale from 1-5 where 1 = strongly disagree and 5 = strongly agree*   1. I have a positive view about medical research in general. 2. Medical researchers can be trusted to protect the interests of people who take part in their studies. 3. We all have some responsibility to help others by volunteering for medical research. 4. Society needs to devote more resources to medical research. 5. Participating in medical research is generally safe. 6. If I volunteer for medical research, I know my personal information will be kept private and confidential. 7. Medical research will find cures for many major diseases during my lifetime. |  |
| **The Revised Healthcare System Distrust Scale** | *Please rate the following statements on a scale from 1-5 where 1 = strongly disagree and 5 = strongly agree*   1. The Health Care System does its best to make patients’ health better 2. The Health Care System covers up its mistakes 3. Patients receive high quality medical care from the Health Care System 4. The Health Care System makes too many mistakes 5. The Health Care System puts making money above patients’ needs 6. The Health Care System gives excellent medical care 7. Patients get the same medical treatment from the Health Care System, no matter what the patient’s race or ethnicity 8. The Health Care System lies to make money 9. The Health Care System experiments on patients without them knowing |  |
| **Demographics** | Age Group, Gender, Race, Ethnicity, Education, Employment, Household Income, Years on Dialysis, Hospitalizations in Past 12 months, Co-Morbidities from the Medical History | Age Group, Gender, Race, Ethnicity, Practice Setting, Practice Size, Clinical Trials ad Investigator/Co-I |

**Appendix B. Patient scores on the pre-test research knowledge questionnaire (N=200)**

| **True or False Questions** | **Correct N (%)** |
| --- | --- |
| The purpose of this research study is to find out which treatment is best. | 190 (95) |
| The treatment that a patient receives is assigned by chance. | 165 (83) |
| Doctors currently don’t know how much to lower blood pressure to help patients the most. | 177 (89) |
| Patients would only be included in the clinical trial if their doctors approve. | 177 (89) |
| Patients get to choose their treatment group. | 158 (79) |

**Appendix C. Physician characteristics**

| **Characteristic** | **Physicians included (n=203)** |
| --- | --- |
| Sex* |  |
| Male | 141 (70%) |
| Female | 61 (30%) |
| Age* |  |
| 30-39 | 74 (37%) |
| 40-49 | 69 (34%) |
| 50-59 | 22 (11%) |
| 60-69 | 27 (13%) |
| Over 70 | 10 (5%) |
| Race* |  |
| White | 114 (56%) |
| Black | 6 (3%) |
| Asian | 79 (39%) |
| Native American | 2 (1%) |
| Hawaiian | 1 (0%) |
| Ethnicity* |  |
| Hispanic | 8 (4%) |
| Not Hispanic | 194 (96%) |
| Fellowship completion year* |  |
| 2010-2014 | 72 (36%) |
| 2005-2009 | 40 (20%) |
| 2000-2004 | 27 (13%) |
| 1990-1999 | 26 (13%) |
| 1980-1989 | 23 (11%) |
| 1970-1979 | 12 (6%) |
| Before | 2 (1%) |
| Practice setting* |  |
| Academic | 82 (41%) |
| Community | 120 (59%) |
| Size of physician practice* |  |
| 1-4 physicians | 73 (36%) |
| 5-9 physicians | 52 (26%) |
| 10 or more physicians | 77 (38%) |
| Participated in a clinical trial as an investigator* |  |
| None | 38 (38%) |
| Yes, 1-2 trials | 28 (28%) |
| Yes, 3-5 trials | 18 (18%) |
| Yes, 6 or more trials | 16 (16%) |

*Only reported for 202 physicians because 1 physician has missing information.

**Appendix D.** **Unadjusted associations between physician characteristics and willingness to participate^a^**

| **Characteristic** | **Participate in trial, n(%)** | **OR (95% CI)** | **p-value** |
| --- | --- | --- | --- |
| Autonomy |  |  |  |
| Low | 494 (61%) | 1.00 | 0.96 |
| High | 493 (61%) | .99 (.77-1.27) |  |
| Burden |  |  |  |
| Low | 491 (60%) | 1.00 | 0.79 |
| High | 496 (61%) | 1.04 (.80-1.34) |  |
| Consent |  |  |  |
| Opt in | 306 (77%) | 1.00 | <0.001 |
| Opt out | 278 (69%) | 0.50 (0.26, 0.96) |  |
| Notification | 253 (62%) | 0.33 (0.18, 0.59) |  |
| No notification | 150 (36%) | 0.04 (0.02, 0.08) |  |
| Sex |  |  |  |
| Male | 703 (62%) | 1.00 | 0.24 |
| Female | 280 (57%) | .74 (.46-1.21) |  |
| Age |  |  |  |
| 30-39 | 385 (65%) | 1.00 | 0.42 |
| 40-49 | 335 (61%) | 0.82 (0.49-1.36) |  |
| 50-59 | 95 (54%) | 0.56 (0.22-1.4) |  |
| Over 60 | 168 (57%) | 0.63 (0.31-1.28) |  |
| Fellowship completion year |  |  |  |
| 2010-2014 | 370 (64%) | 1.00 | 0.67 |
| 2005-2009 | 193 (60%) | 0.83 (0.48-1.44) |  |
| 2000-2004 | 132 (61%) | 0.9 (0.39-2.05) |  |
| 1990-1999 | 126 (61%) | 0.86 (0.36-2.02) |  |
| Before 1990 | 162 (55%) | 0.59 (0.29-1.18) |  |
| Practice setting |  |  |  |
| Academic | 385 (59%) | 1.00 | 0.36 |
| Community | 598 (62%) | 1.24 (.78-2.00) |  |
| Size of physician practice |  |  |  |
| 1-4 physicians | 365 (62%) | 1.00 | 0.27 |
| 5-9 physicians | 228 (55%) | 0.64 (0.21-1.21) |  |
| 10 or more physicians | 390 (63%) | 1.04 (0.28-1.75) |  |
| Participated in a clinical trial as an investigator |  |  |  |
| None | 380 (62%) | 1.00 | 0.51 |
| Any | 603 (60%) | 0.85 (0.53-1.37) |  |

^a^Willingness to participate was defined as “definitely willing” or “probably willing” categories combined
